# Baseline habitual dietary nitrate intake and Alzheimer's Disease related neuroimaging biomarkers in the Australian Imaging, Biomarkers and Lifestyle study of ageing

**DOI:** 10.1016/j.tjpad.2025.100161

**Published:** 2025-04-11

**Authors:** Anjana Rajendra, Nicola P. Bondonno, Kevin Murray, Liezhou Zhong, Stephanie R. Rainey-Smith, Samantha L. Gardener, Lauren C. Blekkenhorst, Vincent Doré, Victor L. Villemagne, Simon M. Laws, Belinda M. Brown, Kevin Taddei, Colin L. Masters, Christopher C. Rowe, Ralph N Martins, Jonathan M. Hodgson, Catherine P. Bondonno, AIBL Research Group

**Affiliations:** a Nutrition & Health Innovation Research Institute, School of Medical and Health Sciences, Edith Cowan University, Perth, Western Australia, Australia; b The Danish Cancer Institute, Copenhagen, Denmark; c School of Population and Global Health, University of Western Australia, Perth, Western Australia, Australia; d Centre for Healthy Ageing, Health Futures Institute, Murdoch University, Murdoch, Western Australia, Australia; e Lifestyle Approaches Towards Cognitive Health Research Group, Murdoch University, Murdoch, Western Australia, Australia; f Centre of Excellence for Alzheimer's Disease Research & Care, School of Medical and Health Sciences, Edith Cowan University, Joondalup, Western Australia, Australia; g Australian Alzheimer's Research Foundation, Nedlands, Western Australia, Australia; h School of Psychological Science, University of Western Australia, Perth, Western Australia, Australia; i Australian E-Health Research Centre, CSIRO, 351 Royal Parade, Parkville, Victoria, Australia; j Department of Molecular Imaging and Therapy, Austin Health, 145 Studley Road, Heidelberg, Victoria, Australia; k Department of Psychiatry, University of Pittsburgh, Thomas Detre Hall, 3811 O'Hara Street, Pittsburgh, PA, USA; l Centre for Precision Health, Edith Cowan University, 270 Joondalup Drive, Joondalup, Western Australia, Australia; m Collaborative Genomics and Translation Group, Edith Cowan University, 270 Joondalup Drive, Joondalup, Western Australia, Australia; n Curtin Medical School, Curtin University, Kent Street, Bentley, Western Australia, Australia; o The Florey Institute, The University of Melbourne, Parkville, Victoria, Australia; p Medical School, The University of Western Australia, Royal Perth Hospital Research Foundation, Perth, Western Australia, Australia; q For a full list of the AIBL Research Group see aibl.org.au

**Keywords:** Nitrate, Alzheimer's disease, Dementia, Cerebral beta-amyloid, Diet, *Apoe*, Neuroimaging brain biomarkers, Brain atrophy

## Abstract

**Background:**

Dietary nitrate, as a nitric oxide (NO) precursor, may support brain health and protect against dementia.

**Objective:**

Our primary aim was to investigate whether dietary nitrate is associated with neuroimaging markers of brain health linked with Alzheimer's disease (AD).

**Participants:**

Study participants were cognitively unimpaired individuals from the Australian Imaging, Biomarkers and Lifestyle Study of Ageing (AIBL) who had β-amyloid positron emission tomography (PET) scans (*n* = 554) and magnetic resonance imaging (MRI) scans (*n* = 335) and had completed a Food Frequency Questionnaire at baseline.

**Methods:**

Source-specific nitrate intakes were estimated using comprehensive nitrate food composition databases. Rates of cerebral β-amyloid (Aβ) deposition, measured using PET, and rates of brain atrophy, measured using MRI, were assessed between baseline and 126-months follow-up, at intervals of 18 months. Multivariable-adjusted linear mixed effect models were used to examine associations between baseline source-specific nitrate intake and rates of (i) cerebral Aβ deposition and (ii) brain atrophy, over the 126 months of follow-up. Analyses were carried out following stratification of the sample by established dementia Alzheimer's disease (AD) risk factors including sex and presence or absence of the apolipoprotein E (APOE) ε4 allele.

**Results:**

In women carriers of the *APOE* ε4 allele, higher plant sourced nitrate intake (median intake 121 mg/day), was associated with a slower rate of cerebral Aβ deposition [β: 4.47 versus 8.99 Centiloid (CL) /18 months, *p* < 0.05] and right hippocampal atrophy [-0.01 versus -0.03 mm3 /18 months, *p* < 0.01], after multivariable adjustments. Moderate intake showed protective associations in men carriers and in both men and women non-carriers of *APOE* ε4.

**Conclusions:**

Associations were observed between plant-derived nitrate intake and cerebral Aβ deposition, particularly in high-risk populations (women and *APOE* ε4 carriers). Associations were also observed for brain volume atrophy, however these exhibited subgroup variability without clear patterns relative to sex and *APOE* ε4 allele carriage. These findings suggest a potential link between plant-sourced nitrate and AD related neuroimaging markers of brain health improved brain health, but further validation in larger studies is required.

## 1 Introduction

Alzheimer's disease (AD) is the most common type of dementia [1]. AD is characterized by histopathological changes in the brain, such as extracellular cerebral β-amyloid (Aβ) deposition and intracellular tau aggregates, along with progressive brain atrophy [2,3]. The 2024 Lancet Commission reported that modifying lifestyle risk factors could prevent or delay up to 40 % of dementia cases [4]. Given the current dearth of effective treatments for dementia and with diet impacting five of the twelve identified modifiable risk factors, identifying beneficial elements of optimal diets to delay or ideally prevent dementia onset is an important prevention strategy.

Beyond the modifiable risk factors, the established non-modifiable risk factors for dementia are age, sex, and genetics [5,6]. A strong genetic risk factor for AD, the most prevalent type of dementia, is carriage of the ε4 allele of the apolipoprotein E (*APOE*) gene [7]. It is therefore necessary to consider the impact of non-modifiable risk factors, such as *APOE* genotype and sex, on any preventative approach including the identification of optimal diets and their protective components. To this end, our previous work has shown that diet can impact cognitive function in an *APOE* genotype contingent manner [8], demonstrating an interplay between modifiable and non-modifiable AD risk factors.

A potential beneficial component of optimal dietary patterns with a high plant food intake is nitrate [9]. The primary dietary sources of nitrate are vegetables (∼70–80 %), meat (∼10–15 %), processed meat (∼ 5 %; nitrate and nitrite are highly regulated preservatives in processed meat products) and drinking water (∼1–10 %; nitrate is considered a contaminant in drinking water) [9]. Nitrate intake is linked with both favourable and adverse health effects which are hypothesised to be source dependent [10]. While nitrate has the potential to form carcinogenic *N*-nitroso compounds (NOCs) [11], it also has the beneficial effect of increasing nitric oxide (NO), a key regulatory molecule in the cardiovascular system [12], central nervous system [13], and cerebrovascular system [14], through an established enterosalivary pathway [15,16]. There is now strong evidence that nitrate, primarily from plant sources, improves cardiovascular health through effects on NO [17]. Some clinical trials investigating the impact of dietary nitrate on cognition and cerebral blood flow show beneficial effects [[18], [19], [20]] but the results remain inconsistent [21,22]. We have previously demonstrated an association of higher habitual plant-sourced nitrate intake with dementia-related mortality in 9149 men and women followed for 25 years in the Australian Diabetes, Obesity and Lifestyle (AusDiab) study [23]. We have also observed better cognitive function with higher habitual plant-sourced nitrate intake, in an *APOE* contingent manner, in 1254 individuals followed for 126 months in the Australian Imaging, Biomarkers, and Lifestyle (AIBL) Study of Ageing, a cohort study focusing on factors that determine the development of Alzheimer's disease [8]. However, the association of nitrate with AD-related neuroimaging markers of brain health remains unexplored.

The primary aim of this study was to investigate the association between habitual intake of plant-sourced nitrate and AD-related neuroimaging biomarkers of brain health, cerebral Aβ burden and brain volume atrophy, in a sample of cognitively unimpaired older adults drawn from the Australian Imaging, Biomarkers, and Lifestyle (AIBL) Study of Ageing. These associations were investigated in the context of the non-modifiable AD risk factors of sex and *APOE* ε4. Secondary aims were to investigate the association between (i) vegetable-sourced nitrate intake, and (ii) animal-sourced nitrate (excluding meat where nitrate and nitrite are allowed food additives), with cerebral Aβ burden and brain volume atrophy. We hypothesised that higher intake of plant-sourced nitrate would be associated with 1) lower rate of cerebral Aβ accumulation, and 2) lower rate of brain volume atrophy (left hippocampal volume, right hippocampal volume, grey matter volume, and white matter volume) over a follow-up period of up to 126 months.

## 2 Methods

### 2.1 Study population

The AIBL Study of Ageing is a longitudinal, prospective, and multicentre study that recruited older adult volunteers who were either cognitively unimpaired, had mild cognitive impairment (MCI), or had AD [24]. Participants (*n* = 1112) were initially recruited between 2006 – 2008 with additional participants (*n* = 1247) recruited from 2011 to enrich the cohort. Further details regarding recruitment, assessment, inclusion, and exclusion criteria are described in Ellis et al. [25] and Fowler et al. [24]. Ethics approval for AIBL was granted by the institutional ethics committees of St Vincent's Hospital, Austin Health, Hollywood Private Hospital, and Edith Cowan University [25,36]. Written informed consent was obtained from individuals prior to study participation.

The current study analysed data from two subsets of participants: i) participants (*n* = 554) who underwent Positron Emission Tomography (PET) to measure cerebral Aβ; and ii) Of 554 participants with PET scans, 335 individuals also had Magnetic Resonance Imaging (MRI) scans to measure brain structure. All included participants were cognitively unimpaired at baseline as determined by a clinical panel following a comprehensive battery of neuropsychological measures, aged 60 years or older, and had completed a food frequency questionnaire (FFQ).

### 2.2 Exposures

At baseline participants completed the Cancer Council of Victoria Food Frequency Questionnaire version 2 (CCVFFQ) to assess habitual food and nutrient intake [26,27]. Participants reported their usual intakes of different food and beverage items over the previous 12 months. The CCVFFQ has been validated in relation to 7-day weighed diet records [28]. Intake of dietary nitrate from different sources where nitrate is naturally present (plant-, vegetable-, and animal-sourced nitrate) and is an allowed additive (processed meat) was assessed using the FFQ and quantified in grams/day (g/d).

#### 2.2.1 Plant- and vegetable- sourced nitrate intake

The nitrate content of all plant-sourced foods (vegetables, fruits, cereals, herbs, spices, pulses, and nuts) was calculated using a comprehensive plant-based food nitrate database to estimate intake of plant-based nitrate. This database includes nitrate values from 304 plant-based foods from 64 countries [29]. As the nitrate content of plant food differs according to country of cultivation, the following strategy was employed. For each vegetable, if three or more references were available in the database for Australia, the median of these values was used. If there were less than three entries in the database for Australia, the median of values for all Oceania (Australia, New Zealand, and surrounding islands) was used. If there were less than three references available for Oceania, the median of values for all countries in the database was used. The estimated quantity of the vegetables and plant-based foods consumed (g/day, g/d) was multiplied by the median nitrate value (mg/g) of each vegetable or plant-based food, respectively. A 50 % reduction factor in the assigned nitrate value was applied to cooked vegetables and plant-based foods to account for the effect of cooking [29]. Total plant-based nitrate and vegetable nitrate consumed per day was calculated by summing the nitrate values of each individual plant-sourced food and vegetable, respectively.

#### 2.2.2 Animal-sourced nitrate intake

Animal-sourced nitrate intake was calculated from red meat, dairy, seafood, and poultry. Processed meat products where nitrate and nitrite is an allowed food additive was not included in this calculation as it has links with negative health effects [30]. For the current study, a recently published animal-sourced nitrate food composition database, with data from 51 countries, was used to calculate animal-sourced nitrate intake [31]. The same strategy for assigning animal-sourced nitrate values was used as described for plant-based foods and vegetables. As there are inadequate data on the impact of cooking on nitrate content of animal-based foods, no reduction factor was applied to account for the effect of cooking. Total animal-sourced nitrate consumed (mg/d) was determined by multiplying the amount of the specific animal-based food consumed (g/d) by its median nitrate content (mg/g).

#### 2.2.3 Total nitrate intake

Total nitrate intake (mg/d) was determined by calculating the sum of nitrate intake values from all food items included in the FFQ including discretionary foods such as chocolate, biscuits, pizza, crisps, and alcohol. Nitrate intake (mg/d) was ascertained by multiplying the amount of food item consumed (g/d) by the assigned median nitrate value (mg/g) for that food item. Food items were assigned a value of zero if the nitrate value for that food item was not available in any of the above listed databases.

### 2.3 Study outcomes

#### 2.3.1 Cerebral aβ positron emission tomography

For each participant included in the current analysis, cerebral Aβ PET imaging was conducted at baseline, and thereafter up to seven follow-up timepoints, 18-months apart. Brain Aβ PET scans were conducted using one of five Aβ-binding ligands (^11^C-Pittsburgh Compound B, ^18^F-Flutemetamol, ^18^F-Florbetaben, ^18^F-Florbetapir, or ^18^F-NAV4694). The CapAIBL image processing software was used to calculate a Centiloid (CL) value for each PET image, standard methods were used to convert specific tracer uptake values to the Centiloid scale, providing a single continuous variable representing brain Aβ burden [32,33]. A CL value of ‘0′ represents the typical Aβ burden in young controls, and ‘100’ the typical Aβ burden seen in mild AD patients [34].

#### 2.3.2 Magnetic resonance imaging

Participants underwent 3D T1-weighted magnetization-prepared rapid acquisition gradient-echo sequence using the following acquisition parameters: slice thickness 1.2 mm, in-plane resolution 1 × 1 mm, repetition time (TR)/echo time (TE)/inversion time (TI) = 2300/2.98/900, flip angle 9 °, and field of view (FOV) 240×256. Scans were then segmented into grey and white matter, cerebrospinal fluid and regions-of-interest using an implementation of the expectation maximization algorithm [35]. The Harmonised Hippocampus Protocol was used for hippocampal extraction [36]. All MRI measures were corrected for total intracranial volume and scanner (multiple scanners are used across study sites). For each participant included in the current analysis, MRI was conducted at baseline, and thereafter up to seven follow-up timepoints, 18-months apart.

### 2.4 Covariates

All demographic data including age, sex (men/women), education level (≤ 12 years and > 12 years), marital status (single/married/divorced/widowed), smoking status (never/former/current), and alcohol intake (yes/no) were self-reported via a questionnaire administered by study staff. Habitual physical activity was self-reported using the long form International Physical Activity Questionnaire, and body mass index (BMI) was calculated as weight in kilograms divided by height in meteres squared with height and weight measured by study personnel [25]. Specific TaqMan® (Thermo Fisher Scientific, Waltham, MA, USA) assays were used to determine Apolipoprotein E (*APOE*) genotype (rs7412, assay ID: C____904,973_10; rs429358, assay ID: C___3,084,793_20) from DNA extracted from fasted blood samples as per standard protocols [37]. Dietary covariates were recorded using CCVFFQ, as mentioned earlier.

### 2.5 Statistical analysis

Statistical analyses were performed using Stata version 15 (StataCorp, College Station, Texas 77,845, USA). Given the different underlying risks of AD, analyses were stratified by *APOE* ε4 allele carrier status. The interaction between sex and dietary nitrate was significant indicating that effects of dietary nitrate may differ in women and men and therefore analyses were stratified by sex.

We had more than 5 % of cases with missing data for the following covariates: BMI, physical activity, smoking status, and alcohol intake status. We ran logistic regression to investigate the missingness mechanisms to determine whether any of the covariate or auxiliary variables can predict probability of missingness in any of the variables as per Rubin's framework [38]. The probability of missingness was associated with educational status and age. We employed multiple imputation (MI) to impute the missing data for variables with more than 5 % missing cases to reduce non-response bias and to improve precision and power [39,40]. Missing values for the covariates of BMI, physical activity, smoking status, and alcohol intake status at baseline were imputed by multivariate imputation by chained equations (MICE), with 20 imputations.

A series of linear mixed effect models were performed using multiple imputation estimations and independent covariance patterns to evaluate the association between habitual nitrate intake separately from different sources (plant-, vegetable-, and animal-sourced, modelled as tertiles) at baseline, and i) cerebral Aβ and ii) brain structure volume, to estimate rate of deposition of cerebral Aβ and rate of brain structure volume atrophy (left hippocampal volume, right hippocampal volume grey matter volume, and white matter volume).

We did not discretely examine associations for meat with nitrate as an allowed additive as it contributed ∼0.45 % to total dietary nitrate intake. Covariates were selected *a priori.* The following models of adjustment were used: Model 1 [age, sex, time (baseline and every 18 month follow-up timepoint until 126 months), interaction term (time by independent variable)], Model 2 [age, sex, time, interaction term (time by independent variable), BMI, physical activity, smoking status, education level, marital status, total energy intake] and Model 3 when plant-sourced nitrate was the exposure of interest [all the covariates adjusted for in Model 2 plus the dietary confounders: intakes of alcohol (yes/no), (g/d) of red meat, fish, saturated fatty acids, polysaturated fatty acids, monosaturated fatty acids] and when naturally occurring animal-sourced nitrate was the exposure of interest, Model 3 included all covariates in Model 2, plus intakes of alcohol (yes/no), (g/d) of saturated fatty acids, polysaturated fatty acids, monosaturated fatty acids and vegetables. All covariates were added as fixed effects, participant as a random effect, and AD-related neuroimaging biomarkers of brain health as the dependent variable.

## 3 Results

### 3.1 Baseline characteristics

Of the analytic cohort, 554 participants underwent PET scans to measure cerebral Aβ and 335 underwent MRI scans to measure brain structure volumes. Study participants who underwent PET scans had a median [IQR] age of 71 [67–75] years at study entry, and a median [IQR] follow-up time of 18 [0–36] months with a maximum follow-up of 126 months. Thirty-two percent were carriers of the *APOE* ε4 allele and just over half the participants were women. The median [IQR] intake of plant-sourced nitrate was 72 [53–100] mg/d, vegetable-sourced nitrate intake was 54 [39–76] mg/d, and animal-sourced nitrate intake was 6 [3–9] mg/d. Of the total nitrate intake, vegetable-sourced nitrate intake contributed 61 %, fruit-sourced nitrate 14 %, whole-grain-sourced nitrate 2 %, animal-sourced nitrate 7 %, meat where nitrate is an allowed additive 0.5 % and discretionary foods 15.5 %. The main contributors to vegetable-sourced nitrate intake were lettuce (30 %), spinach (19 %), beetroot (8 %), potatoes (7 %), and celery (6 %). The primary contributors to animal-sourced nitrate (except processed meat products) in this cohort were yoghurt (55 %), tinned fish (12 %), lamb (9 %), beef (5 %), and grilled fish (4 %). Study participants who underwent MRI scans had a median [IQR] age of 71 [67–76] years at study entry and had similar demographic and dietary characteristics to those participants with PET scans.

Baseline characteristics of the study participants stratified by *APOE* ε4 allele carrier status and sex are presented in Tables 1 **and** 2. In both groups, women carriers of the *APOE* ε4 allele in the highest tertile of plant-sourced nitrate intake, consumed higher vegetable-sourced nitrate and lower amounts of processed-meat sourced nitrate but did not have a higher total vegetable intake compared to the men carriers of the *APOE* ε4 allele, and *APOE* ε4 allele non-carriers (men and women) with the highest plant source nitrate intakes.

**Table 1:** Descriptive statistics of the sample with cerebral β-amyloid data by intake of plant-sourced nitrate.

	*APOE* ε4 Carriers						*APOE* ε4 non-Carriers					
	Women (*n* = 96)			Men (*n* = 83)			Women (*n* = 216)			Men (*n* = 159)		
	Plant-sourced nitrate tertiles			Plant-sourced nitrate tertiles			Plant-sourced nitrate tertiles			Plant-sourced nitrate tertiles		
	T1 *n* = 32	T2 *n* = 32	T3 *n* = 32	T1 *n* = 28	T2 *n* = 28	T3 *n* = 27	T1 *n* = 72	T2 *n* = 72	T3 *n* = 72	T1 *n* = 53	T2 *n* = 53	T3 *n* = 53
**Plant-nitrate intake (mg/day)**	49 [39, 58]	78 [70, 90]	121 [112, 154]	47 [40, 50]	67 [62, 75]	101 [90, 117]	45 [36, 50]	66 [62, 76]	107 [93, 131]	52 [44, 58]	80 [71, 91]	116 [104, 129]
**Vegetable-nitrate intake (mg/day)**	35 [26, 43]	60 [50, 69]	101 [84, 124]	33 [26, 37]	50 [44, 54]	76 [67, 92]	30 [24, 40]	51 [46, 58]	85 [71, 109]	33 [28, 43]	59 [53, 71]	90 [80, 100]
**Animal-nitrate intake (mg/day)**	4 [2, 7]	8 [4, 10]	9.3 [5, 12]	3.9 [3.1, 9.0]	5.8 [3.0, 9.7]	5.8 [4.5, 9.3]	5 [2, 8]	7 [3, 9]	8 [4, 10]	4 [2, 7]	4 [3, 8]	8 [4, 10]
**Processed-meat nitrate intake (mg/day)**	0.21 [0.09, 0.47]	0.25 [0.09, 0.59]	0.15 [0.03, 0.30]	0.46 [0.17, 0.94]	0.47 [0.27, 0.90]	0.52 [0.3, 1.0]	0.26 [0.10, 0.45]	0.19 [0.07, 0.34]	0.19 [0.05, 0.43]	0.47 [0.22, 0.63]	0.46 [0.22, 0.89]	0.44 [0.25, 0.75]
**Total nitrate intake (mg/day)**	60 [49, 71]	99 [88, 105]	136 [124, 169]	69 [59, 74]	93 [80, 105]	124 [107, 134]	58 [51, 65]	82 [75, 89]	127 [108, 151]	70 [61, 78]	103 [92, 110]	143 [129, 159]
**Age (years)**	68 [66, 74]	70 [65, 73]	71 [66, 75]	70 [66, 73]	68 [65, 73]	71 [67, 74]	69 [67, 74]	71 [67, 75]	71 [67, 75]	73 [69, 76]	70 [66, 75]	73 [69, 77]
**BMI**	25 [23, 30]	25 [21, 28]	25 [23, 28]	26 [24, 30]	25 [23, 29]	25 [23, 28]	25 [23, 27]	25 [22, 28]	24 [22, 27]	25 [23, 30]	26 [24, 27]	25 [23, 28]
**MET Score**	3319 [1620, 7860]	4087 [2029,10,050]	4851 [3652, 10,170]	2293 [1130, 4980]	5388 [2871, 7480]	4167 [2874, 7567]	4002 [2034, 6444]	3398 [1908, 6115]	4150 [2616, 6804]	2252 [1222, 3108]	4929 [2184, 7347]	4452 [2874, 8350]
**Education status**												
≤ 12 years	14 (45)	16 (50)	13 (40)	11 (39.2)	9 (32.1)	14 (51)	25 (35)	25 (34)	35 (48)	15 (28)	28 (52)	22 (41)
> 12 years	17 (54)	16 (50)	19 (59)	17 (60.7)	19 (67.8)	13 (48)	46 (64)	47 (65)	37 (51)	38 (71)	25 (47)	31 (58)
**Marital status**												
Single	1 (3.2)	1 (3.1)	3 (9.3)	1 (3.5)	1 (3.5)	1 (3.5)	3 (4.1)	1 (1)	5 (7)	2 (3)	1 (1)	3 (5)
Married	20 (64.5)	19 (59.3)	19 (59.3)	25 (89.2)	26 (92.8)	25 (89.2)	48 (66)	47 (65)	37 (52)	48 (90)	47 (90)	45 (84)
Divorced/separated	5 (16.1)	6 (18.7)	5 (15.6)	1 (3.5)	1 (1)	1 (3.7)	15 (20)	12 (16)	14 (19)	1 (1)	1 (1)	4 (7)
Widowed	5 (16.1)	6 (18.7)	5 (15.6)	1 (3.5)	0 (0)	0 (0)	6 (8)	12 (16)	15 (21)	2 (3)	3 (5)	1 (1)
**Smoking status**												
Never	16 (72)	16 (69.5)	14 (58.3)	14 (63.6)	12 (66.6)	14 (58.3)	37 (68)	36 (66)	41 (75)	23 (60)	21 (48)	20 (52)
Former	6 (27)	5 (21.7)	8 (33.3)	7 (31.8)	6 (33.3)	10 (41.6)	13 (24)	16 (29)	13 (24)	14 (36)	18 (41)	16 (42)
Current	0 (0)	2 (8)	2 (8.3)	1 (4.5)	0 (0)	0 (0)	4 (7)	2 (3)	0 (0)	1 (2)	4 (9)	2 (5)
**High brain Aβ (Centiloid ≥ 20)**	17 (53)	13 (40)	9 (28)	9 (31.0)	13 (46.4)	19 (70)	8 (11)	8 (11)	12 (16)	9 (16)	10 (18)	8 (15)
**Brain Aβ (Centiloid)**	32 [−2, 66]	7 [−2., 56]	9.1 [−3, 53]	12.6 [−3.8, 51]	16 [−2.05, 85]	50 [17, 92]	1.6 [−3.3, 14]	−1 [−4, 10]	1.6 [−3, 11]	−0.3 [−3, 8]	4.3 [−2, 14]	0.4 [−3, 17]
**Dietary characteristics**												
Energy (kj/d)	4680 [3378, 5834]	6179 [5055, 7270]	6176 [5158, 7358]	6977 [5160, 8523]	7028 [5951, 8871]	9183 [7430, 11,142]	5484 [4696, 6294]	5654 [4678, 6846]	6255 [5246, 7621]	6306 [5037, 7340]	7378 [6231, 9093]	8269 [6647, 9787]
Total fish intake (g/day)	17 [11, 35]	35 [19, 63]	55 [32, 71]	25 [15, 45]	30 [14, 49]	41 [26, 87]	21 [14, 40]	23 [16, 35]	30 [17, 57]	20 [14, 38]	34 [20, 54]	45 [26, 67]
Red meat intake (g/day)	31 [17, 51]	35 [22, 56]	43 [23, 57]	62 [37, 72]	55 [39, 78]	89 [60, 133]	31 [18, 58]	32 [18, 51]	47 [22, 69]	44 [22, 70]	58 [33, 94]	77 [36, 102]
Processed-meat intake (g/day)	8 [2, 18]	7 [3, 22]	5 [1, 12]	15 [6, 36]	15 [10, 34]	19 [12, 36]	9 [3, 15]	6 [2, 12]	7 [2, 17]	13 [5, 21]	16 [8, 30]	16 [8, 30]
Dietary fibre intake (g/day)	14 [10, 18]	18 [16, 26]	21 [17, 27]	18 [16, 21]	22 [18, 27]	27 [22, 34]	14 [12, 19]	19 [15, 22]	22 [17, 28]	19 [15, 21]	23 [20, 28]	29 [22, 33]
Saturated FA (g/day)	18 [13, 22]	21 [17, 31]	20 [12, 28]	27 [19, 38]	24 [20, 33]	34 [28, 43]	22 [17, 27]	20 [16, 28]	23 [18, 30]	26 [17, 31]	26 [17, 36]	31 [24, 43]
Polyunsaturated FA (g/day)	7 [4, 9]	9 [6, 13]	9 [7, 13]	10 [7, 12]	12 [6, 15]	12 [10, 19]	7 [5, 9]	8 [5, 10]	9 [6, 13]	9 [6, 11]	10 [7, 14]	13 [9, 16]
Monosaturated FA (g/day)	16 [12, 21]	20 [17, 25]	20 [17, 26]	23 [18, 31]	22 [19, 31]	33 [23, 44]	18 [14, 22]	19 [15, 24]	22 [17, 29]	21 [16, 27]	24 [19, 29]	28 [24, 37]
Fruit intake (g/day)	191 [116, 245]	250 [189, 353]	343 [241, 424]	152 [100, 194]	271 [189, 373]	396 [322, 531]	182 [94, 252]	243 [124, 346]	333 [219, 433]	232 [112, 337]	306 [154, 399]	355 [255, 493]
Vegetable intake (g/day)	107 [80, 135]	150 [119, 173]	189 [151, 236]	100 [86, 144]	164 [131, 198]	207 [178, 264]	95 [72, 119]	128 [111, 170]	203 [161, 240]	115 [87, 143]	156 [130, 191]	236 [190, 286]
Alcohol intake (g/day)	58 [14, 151]	108 [45, 222]	23 [4, 139]	85 [275, 338]	220 [74, 531]	244 [77, 352]	87 [21, 211]	61 [5, 146]	47 [9, 191]	135 [35, 348]	196 [56, 378]	156 [60, 346]

Median [IQR], n ( %). Abbreviations: Aβ, amyloid beta; *APOE*, Apolipoprotein E (gene); BMI, body mass index calculated as weight in kilograms divided by height in meters squared; FA, fatty acids; IQR, interquartile range; MET, metabolic equivalent of task, unit of habitual physical activity derived from the International Physical Activity Questionnaire; g/day, grams per day; kj/day, kilojoules per day; n, number; mg/day, milligrams per day; %, percentage; T, tertile.

**Table 2:** Descriptive statistics of the sample with MRI biomarkers of brain health by intake of plant-sourced nitrate.

	*APOE* ε4 Carriers						*APOE* ε4 non-Carriers					
	Women (*n* = 64)			Men (*n* = 56)			Women (*n* = 123)			Men (*n* = 92)		
	Plant-sourced nitrate tertiles			Plant-sourced nitrate tertiles			Plant-sourced nitrate tertiles			Plant-sourced nitrate tertiles		
	T1 *n* = 22	T2 *n* = 21	T3 *n* = 21	T1 *n* = 19	T2 *n* = 19	T3 *n* = 18	T1 *n* = 41	T2 *n* = 41	T3 *n* = 41	T1 *n* = 31	T2 *n* = 31	T3 *n* = 30
**Plant-nitrate intake (mg/day)**	49 [39, 58]	82 [72, 90]	119 [103, 150]	43 [36, 51]	69 [60, 74]	98 [91, 113]	43 [34, 49]	65 [59, 74]	102 [89, 128]	47 [42, 54]	73 [68, 83]	116 [101, 129]
**Vegetable-nitrate intake (mg/day)**	35 [26, 44]	62 [59, 68]	101 [80, 118]	31 [24, 37]	48 [41, 59]	73 [66, 81]	28 [24, 39]	47 [42, 55]	79 [68, 104]	31 [27, 41]	54 [47, 60]	87 [80, 98]
**Animal-nitrate intake (mg/day)**	5 [2, 9]	8 [5, 12]	9 [7, 12]	3.6 [2.3, 8.0]	5.0 [3.1, 9.4]	6.6 [4.5, 9.0]	5.0 [2.9, 8.5]	6.7 [2.8, 9.6]	8.9 [4.6, 10.2]	3.6 [2.4, 6.4]	4.8 [3.3, 7.3]	6.9 [4.1, 11.0]
**Processed-meat nitrate intake (mg/day)**	0.34 [0.09, 0.89]	0.26 [0.08, 0.57]	0.11 [0.01, 0.22]	0.56 [0.14, 0.93]	0.44 [0.24, 0.93]	0.47 [0.34, 1.01]	0.31 [0.09, 0.44]	0.19 [0.11, 0.40]	0.18 [0.04, 0.45]	0.2 [0.1, 0.6]	0.4 [0.1, 0.9]	0.6 [0.4, 1.1]
**Total nitrate intake (mg/day)**	63 [52, 73]	102 [99, 111]	140 [127, 173]	65 [54, 75]	93 [81, 103]	124 [111, 133]	58 [47, 64]	81 [75, 85]	128 [106, 155]	68 [61, 77]	99 [89, 108]	144 [127, 159]
**Age (years)**	67 [65, 74]	69 [66, 72]	70 [66, 75]	70 [67, 74]	67 [65, 71]	71 [67, 74]	69 [67, 77]	72 [67, 75]	73 [69, 77]	74 [67, 78]	72 [67, 79]	74 [71, 76]
**BMI**	25 [22, 39]	26 [23, 28]	25 [23, 26]	26 [24, 30]	25 [23, 27]	25 [23, 28]	26 [23, 28]	25 [22, 27]	25 [22, 27]	24 [27, 30]	26 [24, 27]	25 [24, 28]
**MET Score**	3798 [1608,9267]	5577 [2412, 10,839]	3725 [2787, 9279]	2298 [1464, 4980]	4600 [2886, 7417]	4167 [2847, 7956]	3072 [1786, 5564]	3512 [2176, 4566]	4150 [2448, 6655]	2373 [1176, 4122]	3150 [1983, 7347]	5586 [3405, 9324]
**Education status**												
≤ 12 years	12 (54)	13 (61)	8 (38)	8 (42)	6 (31.5)	9 (50)	13 (32)	18 (43)	21 (51)	12 (38)	19 (61)	13 (43)
> 12 years	10 (45)	8 (38)	13 (61)	11 (57)	13 (68.4)	9 (50)	27 (67)	23 (56)	20 (48)	19 (61)	12 (38)	17 (56)
**Marital status**												
Single	1 (4.5)	0 (0)	3 (14.2)	1 (5.2)	1 (5.2)	1 (5.5)	2 (4)	0 (0)	5 (12)	1 (3)	1 (3)	2 (6)
Married	12 (54.5)	15 (71.4)	11 (52.3)	17 (89.4)	17 (89.4)	16 (88.8)	23 (56)	29 (70)	17 (41)	28 (90)	28 (90)	24 (80)
Divorced/separated	4 (18.1)	3 (14.2)	4 (19.0)	1 (5.2)	1 (5.2)	1 (5.5)	10 (24)	6 (14)	7 (17)	0 (0)	0 (0)	4 (13)
Widowed	5 (22.7)	3 (14.2)	3 (14)	0 (0)	0 (0)	0 (0)	6 (14)	6 (14)	12 (29)	2 (6)	2 (6)	0 (0)
**Smoking status**												
Never	10 (62.5)	13 (72.2)	11 (57.8)	11 (64.7)	12 (75.0)	10 (58.8)	20 (57)	25 (64)	25 (65)	14 (46)	14 (45)	11 (42)
Former	6 (37.5)	4 (22.2)	6 (31.5)	5 (29.4)	4 (25.0)	7 (41.6)	12 (34)	14 (35)	13 (34)	15 (50)	14 (45)	13 (50)
Current	0 (0)	1 (5)	2 (10.5)	1 (5.8)	0 (0)	0 (0)	3 (8)	0 (0)	0 (0)	1 (3)	3 (9)	2 (7)
**High brain Aβ (Centiloid ≥ 20)**	15 (68.1)	12 (57.1)	8 (38.1)	8 (42)	12 (63)	15 (83)	8 (19)	4 (9)	11 (26)	7 (22)	7 (22)	7 (23)
**Brain Aβ (Centiloid)**	47 [10.8, 75.4]	35.1 [8.1, 62]	12.6 [−1.5, 60]	15.9 [−1.8, 82]	67 [2, 102]	66 [31, 93]	3. [−3, 17]	−1 [−2, 12]	3.8 [−0.9, 27]	−0.1 [−2, 31]	7 [−2, 26]	1 [−1, 18]
**Dietary characteristics**												
Energy (kj/d)	4766 [3378, 5955]	5783 [5199, 8374]	6257 [5026, 7152]	7149 [5379, 8749]	6717 [5859, 9064]	9645 [6585, 11,463]	5484 [4696, 5948]	5812 [5044, 7265]	6966 [53,582, 7931]	6386 [5253, 7340]	6958 [5942, 9122]	8499 [7012, 10,758]
Total fish intake (g/day)	24 [12, 50]	49 [25, 68]	36 [16, 67]	27 [16, 51]	40 [13, 59]	39 [26, 98]	21 [13, 43]	23 [13, 34]	37 [18, 58]	20 [16, 46]	33 [20, 52]	47 [34, 65]
Red meat intake (g/day)	30 [16, 49]	37 [22, 69]	42 [19, 57]	62 [30, 77]	58 [41, 85]	94 [60, 140]	41 [22, 62]	31 [11, 51]	51 [19, 76]	49 [30, 85]	51 [21, 104]	89 [43, 122]
Processed-meat intake (g/day)	11 [3, 26]	11 [3, 23]	3.8 [0.6, 6]	19 [5, 33]	16 [9, 35]	17 [12, 35]	10 [3, 14]	7 [3, 13]	6 [1, 17]	10 [5, 21]	16 [6, 30]	22 [14, 39]
Dietary fibre intake (g/day)	14 [11, 18]	21 [17, 29]	20 [15, 26]	18 [16, 21]	24 [19, 28]	29 [20, 34]	13 [12, 19]	20 [16, 23]	24 [18, 31]	18 [14, 21]	25 [21, 30]	30 [24, 33]
Saturated FA (g/day)	18 [13, 22]	22 [17, 34]	19 [12, 25]	25 [19, 38]	26 [20, 36]	30 [23, 43]	20 [16, 24]	20 [17, 28]	24 [19, 31]	27 [16, 33]	23 [17, 36]	31 [26, 46]
Polyunsaturated FA (g/day)	7 [5, 8]	11 [7, 13]	9 [7, 12]	11 [8, 13]	12 [9, 15]	12 [10, 20]	6 [5, 11]	8 [6, 11]	10 [6, 13]	9 [6, 12]	11 [7, 15]	12 [8, 17]
Monosaturated FA (g/day)	17 [12, 23]	21 [17, 25]	19 [15, 25]	20 [18, 32]	28 [19, 33]	31 [22, 44]	18 [14, 21]	21 [17, 25]	24 [16, 32]	21 [16, 27]	24 [18, 29]	28 [24, 41]
Fruit intake (g/day)	197 [138, 256]	268 [196, 364]	353 [218, 439]	152 [103, 271]	271 [201, 388]	395 [325, 523]	182 [94, 266]	296 [185, 358]	359 [253, 455]	232 [112, 334]	226 [167, 412]	355 [279, 510]
Vegetable intake (g/day)	106 [82, 134]	160 [129, 187]	186 [151, 237]	100 [80, 145]	160 [127, 201]	200 [168, 257]	92 [69, 119]	129 [110, 173]	210 [172, 250]	114 [84, 135]	171 [134, 202]	244 [207, 307]
Alcohol intake (g/day)	54 [14, 143]	106 [64, 228]	25 [4, 150]	150 [66, 338]	288 [120, 570]	244 [5, 407]	88 [15, 288]	57 [5, 141]	24 [9, 128]	184 [46, 348]	150 [68, 336]	229 [60, 353]

Median [IQR], n ( %). Abbreviations: Aβ, amyloid beta; *APOE*, Apolipoprotein E (gene); BMI, body mass index calculated as weight in kilograms divided by height in meters squared; FA, fatty acids; IQR, interquartile range; MET, metabolic equivalent of task, unit of habitual physical activity derived from the International Physical Activity Questionnaire; g/day, grams per day; kj/day, kilojoules per day; n, number; mg/day, milligrams per day; %, percentage; T, tertile.

### 3.2 Longitudinal analyses of source-specific nitrate intake and AD-related neuroimaging biomarkers of brain health

Over up to 126 months of follow-up, brain cerebral Aβ deposition increased and brain structure volumes (left hippocampal volume, right hippocampal volume, grey matter volume, and white matter volume) decreased in the whole cohort. Significant interactions were observed between baseline dietary nitrate intake and time for each outcome that was associated with dietary nitrate intake. This indicates differential rates of decline in brain structure volumes and deposition of cerebral Aβ between high, moderate, and low intakes of nitrate from different sources. As such, all results described below are from models with the interaction term included.

#### 3.2.1 Source-specific nitrate intake and cerebral aβ deposition in men and women *APOE* ε4 allele carriers

**Fig. 1:**
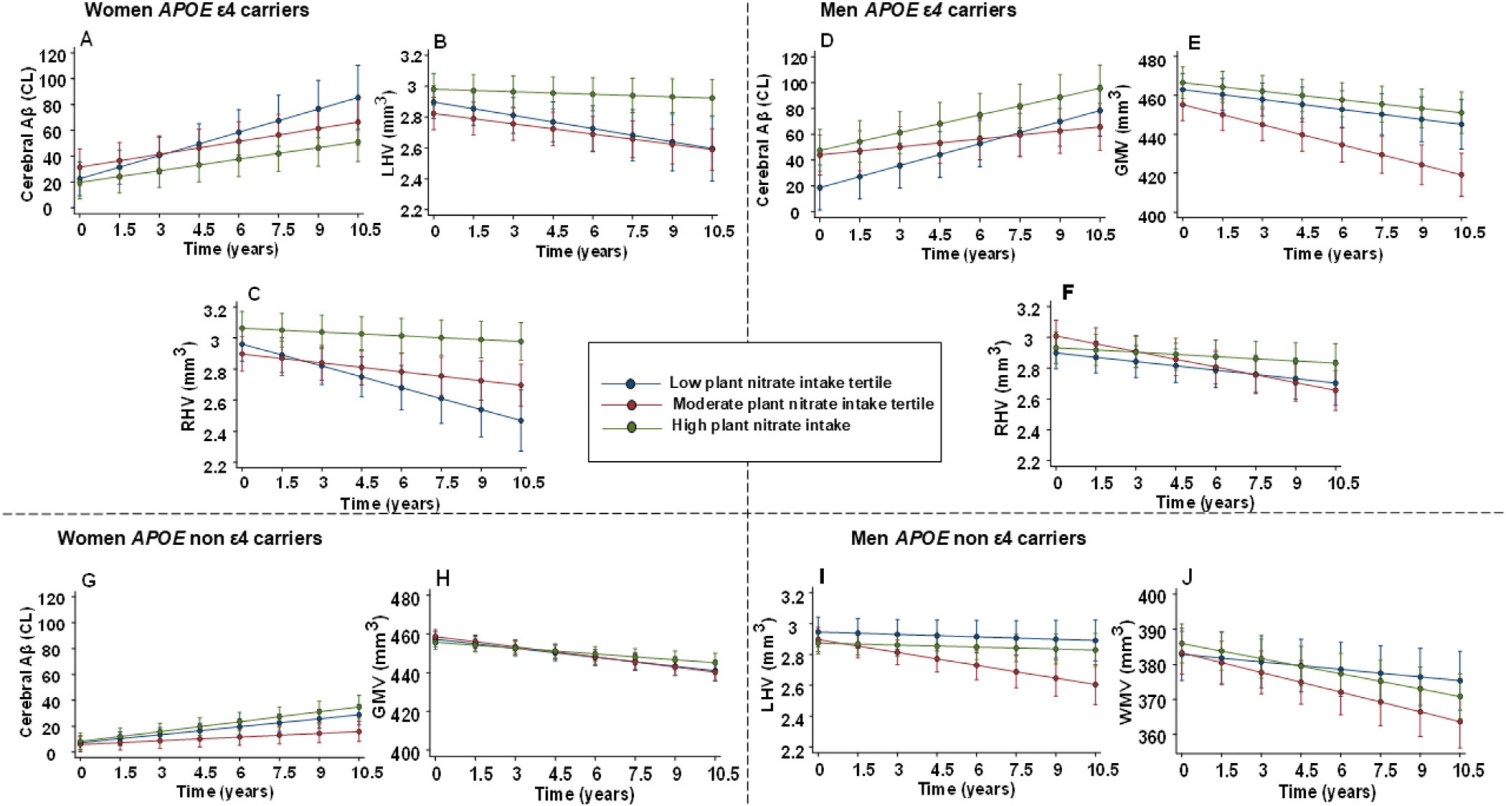
**Trajectories of AD-related neuroimaging biomarkers of brain health by plant-sourced nitrate intake tertiles.** Interaction plots describing the associations between intakes of plant-sourced nitrate and rates of deposition of cerebral beta-amyloid and decline in volume of MRI-based biomarkers of AD in the Australian Imaging, Biomarkers and Lifestyle study of ageing followed up for 10.5 years. Plots based on linear mixed effects models adjusted for age, time, interaction term [time by independent variable (dietary nitrate)], physical activity levels, level of education, body mass index, smoking status, energy intake, marital status [Model 2]. Abbreviations: AD, Alzheimer's disease; *APOE*, Apolipoprotein E (gene); Aβ, beta-amyloid; CL, Centiloid; mm^3^ cubic millimetres; LHV, Left hippocampal volume; RHV, Right hippocampal volume; GMV, Grey matter volume; WMV, White matter volume.

In women *APOE* ε4 allele carriers, participants in the highest tertile of plant-sourced nitrate intake had a significantly lower rate of cerebral Aβ deposition compared to those in the lowest intake tertile (Model 2; Table 3 **and** Fig. 1**A**). A similar association was observed for vegetable-sourced nitrate intake (Model 2; **Supplementary Table 1** and **Supplementary Figure 1A**). No significant association was observed for animal-sourced nitrate intake and rate of cerebral Aβ deposition.

**Table 3:** Linear mixed model associations between sources of nitrate intake and cerebral Aβ burden.

	*APOE* ε4 Carriers						*APOE* ε4 non-Carriers					
	Women			Men			Women			Men		
Plant-sourced nitrate intake	T1 *n* = 32	T2 *n* = 32	T3 *n* = 32	T1 *n* = 28	T2 *n* = 28	T3 *n* = 27	T1 *n* = 72	T2 *n* = 72	T3 *n* = 72	T1 *n* = 53	T2 *n* = 53	T 3n=53
Intake, mg/d	49 (39, 58)	78 (70, 90)	121 (112, 154)	47 (40, 49)	67 (62, 75)	100 (90, 116)	46 (37, 51)	71 (63, 78)	108 (94, 133)	52 (44, 56)	78 (70, 90)	116 (104, 129)
Model 1	8.92 [5.71, 12.13]	5.02* [3.23, 6.81]	4.51* [3.18, 5.85]	8.53 [6.95, 10.11]	3.16⁎⁎⁎ [1.72, 4.59]	6.59 [1.72, 4.59]	3.75 [2.91, 4.59]	1.55⁎⁎⁎ [0.77, 2.34]	3.31 [2.48, 4.15]	1.61 [0.72, 2.50]	1.60 [0.77, 2.42]	2.28 [1.47, 3.10]
Model 2	8.99 [5.81, 12.17]	4.99* [3.23, 6.75]	4.47* [3.14, 5.80]	8.53 [6.94, 10.13]	3.10⁎⁎⁎ [1.52, 4.67]	6.89 [5.58, 8.19]	3.88 [3.04, 4.73]	1.71⁎⁎⁎ [0.91, 2.50]	3.52 [2.67, 4.36]	1.61 [0.72, 2.49]	1.50 [0.68, 2.33]	2.09 [1.26, 2.93]
Model 3	8.95 [5.76, 12.13]	5.00* [3.25, 6.76]	4.50* [3.15, 6.76]	8.56 [6.95, 10.17]	3.11⁎⁎⁎ [1.53, 4.68]	6.87 [5.56, 8.17]	3.88 [3.03, 4.73]	1.70⁎⁎⁎ [0.91, 2.50]	3.51 [2.66, 4.36]	1.62 [0.73, 2.50]	1.51 [0.68, 2.34]	2.10 [1.26, 2.93]
**Vegetable -sourced nitrate intake**												
Intake (mg/d)	35 (26, 43)	60 (55, 68)	101 (84, 124)	32 (26, 37)	50 (46, 54)	75 (67, 91)	30 (24, 37)	51 (46, 57)	85 (72, 109)	33 (27, 40)	58 (51, 67)	90 (82, 100)
Model 1	7.46 [5.13, 9.79]	5.76 [4.01, 7.50]	3.74* [2.29, 5.19]	7.14 [5.71, 8.58]	2.67⁎⁎⁎ [1.07, 4.27]	7.26 [5.95, 8.57]	3.37 [2.49, 4.25]	1.81⁎⁎ [1.08, 2.54]	3.78 [2.88, 4.67]	1.66 [0.79, 2.53]	1.94 [1.11, 2.77]	1.91 [1.09, 2.73]
Model 2	7.56 [5.29, 9.84]	5.69 [3.98, 7.40]	3.70* [2.25, 5.16]	7.63 [6.16, 9.10]	2.58⁎⁎⁎ [0.91, 4.25]	7.52 [6.17, 8.86]	3.53 [2.64, 4.42]	1.97⁎⁎ [1.23, 2.71]	3.93 [3.03, 4.83]	1.63 [0.76, 2.51]	1.77 [0.93, 2.62]	1.80 [0.97, 2.63]
Model 3	7.54 [5.26, 9.81]	5.70 [3.99, 7.41]	3.74⁎⁎ [2.25, 5.22]	7.63 [6.15, 9.11]	2.59⁎⁎⁎ [0.92, 4.26]	7.50 [6.15, 8.85]	3.52 [2.63, 4.41]	1.96⁎⁎ [1.23, 2.70]	3.93 [3.03, 4.83]	1.65 [0.78, 2.52]	1.77 [0.92, 2.62]	1.80 [0.97, 2.62]
**Animal-sourced nitrate intake**												
Intake (mg/d)	2 (1, 3)	7 (5, 8)	12 (10, 16)	2 (2, 3)	5 (4, 6)	10 (9, 11)	2 (1, 3)	7 (5, 8)	10 (9, 12)	2 (1, 3)	5 (4, 6)	10 (9, 11)
Model 1	6.35 [4.33, 8.37]	3.63 [177, 5.49]	5.35 [3.83, 6.87]	7.04 [5.24, 8.83]	5.30 [3.99, 6.60]	6.20 [4.63, 7.77]	2.77 [1.98, 3.56]	3.86 [3.08, 4.65]	1.52* [0.63, 2.41]	1.61 [0.74, 2.48]	2.53 [1.71, 3.35]	1.36 [0.53, 2.18]
Model 2	6.22 [4.21, 8.23]	3.89 [2.06, 5.71]	5.28 [3.75, 6.82]	7.82 [5.88, 9.75]	5.65 [4.32, 6.98]	6.31 [4.67, 7.96]	2.93 2.13, 3.73]	4.05 [3.25, 4.85]	1.67* [0.77, 2.57]	1.61 [0.74, 2.48]	2.53 [1.71, 3.35]	1.36 [0.53, 2.18]
Model 3	6.26 [4.24, 8.28]	3.87 [2.04, 5.70]	5.32 [3.76, 6.88]	7.80 [5.86, 9.74]	5.63 [4.30, 6.97]	6.31 [4.66, 7.97]	2.95 [2.12, 3.72]	4.05 [3.25, 4.85]	1.66* [0.77, 2.56]	1.56 [0.69, 2.43]	2.49 [1.66, 3.31]	1.13 [0.28, 1.97]

Slopes (Centiloid/1.5 years) and 95 % Confidence Intervals were obtained from linear mixed models with the exposure fitted as tertiles. Model 1 adjusted for age, time, interaction term [time by independent variable (dietary nitrate)]; Model 2 adjusted for all covariates in Model 1 plus physical activity levels, level of education, body mass index, smoking status, energy intake, marital status; Model 3 adjusted for all covariates in Model 2 excluding energy intake; when plant- and vegetable- sourced nitrate were the exposures of interests, plus intake (yes/no) of alcohol, (g/d) of red meat, fish, processed meat, saturated fatty acids, polyunsaturated fatty acids, and monounsaturated fatty acids; when naturally occurring animal nitrate was an exposure of interest. Model 3 adjusted for intake (yes/no) of alcohol, (g/d) of vegetables, saturated fatty acids, polyunsaturated fatty acids, and monosaturated fatty. Abbreviations: Aβ, amyloid beta; *APOE*, Apolipoprotein E (gene); n, number; mg/d, milligrams per day; median (Interquartile range); g/day, grams per day.

⁎ , significant interaction between time and independent variable (*p* < 0.05).

⁎⁎ , significant interaction between time and independent variable (*p* < 0.01).

⁎⁎⁎ , significant interaction between time and independent variable (*p* < 0.001) in comparison of slopes of tertile 2 and 3 to tertile 1.

In men *APOE* ε4 allele carriers, significantly lower rates of cerebral Aβ deposition were only observed for participants in the moderate intake tertile of plant-sourced nitrate and vegetable-sourced nitrate (Model 2; Table 3 and Fig. 1**D,** Supplementary **Table 1** & Supplementary **Figure 1C**).

#### 3.2.2 Source-specific nitrate intake and cerebral aβ deposition in men and women *APOE* ε4 allele non-carriers

In women *APOE* ε4 allele non-carriers, participants in the moderate intake tertiles of plant-sourced and vegetable-sourced nitrate had a significantly lower rate of cerebral Aβ deposition compared to those in the lowest and highest intake tertile (Model 2; Table 3 and Fig. 1**G, Supplementary Table 1** and **Supplementary Figure 1F**). Unlike women *APOE* ε4 allele carriers, women non-carriers in the moderate intake tertile of animal-sourced nitrate had a significantly higher rate of cerebral Aβ deposition compared to those in the lowest and highest intake tertile (Model 2; **Supplementary Table 2** and **Supplementary Figure 2D**).

In men *APOE* ε4 allele non-carriers, no significant associations were observed between the intake of nitrate from any source and rate of cerebral Aβ deposition.

#### 3.2.3 Source of nitrate and brain structure volumes in men and women *APOE* ε4 allele carriers

In women *APOE* ε4 allele carriers, compared to those in the lowest intake tertile, participants in the highest tertile of plant-sourced nitrate intake had significantly lower rates of right hippocampal atrophy and those in the moderate intake tertile had significantly lower rates of left hippocampal atrophy. Participants in the highest tertile of vegetable-sourced nitrate intake had significantly lower rates of right hippocampal atrophy and participants in the highest tertile of animal-sourced nitrate intake had significantly lower rates of left and right hippocampal atrophy and grey matter atrophy (Model 2; Table 4 and Fig. 1**B &** 1**C, Supplementary Table 1, Supplementary Figure 1B, Supplementary Table 2** and **Supplementary Figure 2A, 2B, & 2C**).

**Table 4:** Linear mixed model associations between plant-sourced nitrate intake and MRI-based brain volumes.

	*APOE* ε4 Carriers						*APOE* ε4 non-Carriers					
	Women			Men			Women			Men		
Plant-sourced nitrate	T1 *n* = 22	T2 *n* = 21	T3 *n* = 21	T1 *n* = 19	T2 *n* = 19	T3 *n* = 18	T1 *n* = 41	T2 *n* = 41	T3 *n* = 41	T1 *n* = 31	T 2 *n* = 31	T3 *n* = 30
**Intake (mg/d)**	49 [39, 58]	82 [72, 90]	119 [103, 150]	43 [36, 51]	69 [60, 74]	98 [91, 113]	43 [34, 49]	65 [59, 74]	102 [89, 128]	47 [42, 54]	73 [68, 83]	116 [101, 129]
**Left hippocampal volume**												
Model 1	−0.02 [−0.03, −0.00]	−0.04* [−0.06, −0.03]	−0.00 [−0.01, 0.00]	−0.02 [−0.04, −0.00]	−0.03 [−0.05, −0.02]	- 0.02 [−0.04, −0.01]	−0.01 [−0.02, −0.01]	−0.01 [−0.02, −0.01]	−0.01 [−0.02, −0.00]	−0.00 [−0.02, 0.00]	−0.04⁎⁎ [−0.05, −0.02]	−0.00 [−0.01, 0.00]
Model 2	−0.02 [−0.03, −0.02]	−0.04* [−0.06, −0.03]	−0.00 [−0.01, 0.00]	−0.02 [−0.04, −0.01]	−0.04 [−0.05, −0.02]	- 0.02 [−0.04, −0.01]	−0.01 [−0.02, −0.01]	−0.01 [−0.02, −0.01]	−0.01 [−0.02, −0.00]	−0.00 [−0.02, 0.00]	−0.04⁎⁎ [−0.05, −0.02]	−0.00 [−0.01, 0.00]
Model 3	−0.02 [−0.03, −0.00]	−0.04* [−0.06, −0.03]	−0.00 [−0.01, 0.00]	−0.02 [−0.04, −0.00]	−0.04 [−0.05, −0.02]	−0.02 [−0.04, 0.01]	−0.01 [−0.01, −0.00]	−0.01 [−0.02, −0.01]	−0.01 [−0.02, −0.00]	−0.00 [−0.02, 0.00]	−0.04⁎⁎ [−0.05, −0.02]	−0.00 [−0.02, 0.00]
**Right hippocampal volume**												
Model 1	−0.03 [−0.05, −0.02]	−0.03 [−0.05, −0.02]	−0.01⁎⁎ [−0.02, −0.00]	−0.02 [−0.04, −0.00]	−0.04 [−0.05, −0.03]	−0.01 [−0.02, −0.00]	−0.01 [−0.01, −0.00]	−0.01 [−0.02, −0.01]	−0.01 [−0.02, −0.01]	−0.01 [−0.01, −0.00]	−0.02 [−0.02, −0.01]	−0.01 [−0.01, −0.00]
Model 2	−0.03 [−0.05, −0.02]	−0.03 [−0.05, −0.02]	−0.01⁎⁎ [−0.02, −0.00]	−0.02 [−0.04, −0.01]	−0.05* [−0.06, −0.03]	−0.01 [−0.02, −0.00]	−0.01 [−0.01, −0.00]	−0.01 [−0.02, −0.01]	−0.01 [−0.02, −0.01]	−0.01 [−0.01, −0.00]	−0.01 [−0.02, −0.01]	−0.01 [0.02, −0.00]
Model 3	−0.03 [−0.05, −0.02]	−0.03 [−0.05, −0.02]	−0.01* [−0.02, −0.00]	−0.02 [−0.04, −0.01]	−0.05* [−0.06, −0.03]	−0.01 [−0.02, 0.00]	−0.01 [−0.01, −0.00]	−0.01 [−0.02, −0.01]	−0.01 [−0.02, −0.01]	−0.01 [−0.01, −0.00]	−0.01 [−0.02, −0.01]	−0.01 [0.02, −0.00]
**Grey matter volume**												
Model 1	−3.41 [−4.80, −2.01]	−4.36 [−5.70, −3.02]	−2.10 [−2.94, −3.02]	−2.54 [−4.06, −1.01]	−4.98* [−6.13, −3.83]	−2.18 [−3.36, −1.00]	−2.31 [−2.88, −1.73]	−2.56 [−3.02, −2.10]	−1.46* [−2.00, −0.91]	−1.46 [−2.20, −0.72}	−1.78 [−2.58, −0.97]	−1.27 [−1.89, −0.65]
Model 2	−3.36 [−4.76, −1.97]	−4.49 [−5.87, −3.12]	−2.12 [−2.98, −1.26]	−2.54 [−4.12, −0.95]	−5.14⁎⁎ [−6.41, −3.88]	−2.20 [−3.38, −1.02]	−2.32 [−2.90, −1.74]	−2.61 [−3.08, −2.14]	−1.50* [−2.05, −0.95]	−1.47 [−2.22, −0.73]	−1.82 [−2.64, −1.01]	−1.35 [−1.99, −0.70]
Model 3	−3.30 [−4.68, −1.91]	−4.58 [−5.95, −3.21]	−2.18 [−3.04, −1.31]	−2.56 [−4.16, −0.97]	−5.17⁎⁎ [−6.43, −3.91]	−2.16* [−3.34, −0.99]	−2.31 [−2.89, −1.74]	−2.61 [−3.08, −2.14]	−1.50* [−2.05, −0.95]	−1.47 [−2.21, −0.73]	−1.82 [−2.63, −1.00]	−1.35 [−2.00, −0.71]
**White matter volume**												
Model 1	−3.40 [−4.42, −2.37]	−2.41 [−3.39, −1.42]	−2.22 [−2.84, −1.61]	−2.24 [−3.39, −1.08]	−2.57 [−3.44, −1.70]	−2.68 [−3.58, −1.79]	−1.26 [−1.79, −0.74]	−1.21 [−1.63, −0.79]	−1.27 [−1.77, −0.77]	−1.10 [−1.76, −0.45]	−2.84⁎⁎⁎ [−3.55, −2.13]	−2.20 [−2.75, −1.65]
Model 2	−3.39 [−4.39, −2.38]	−2.34 [−3.34, −1.34]	−2.30 [−2.92, −1.68]	−2.35 [−3.55, −1.14]	−2.36 [−3.31, −1.41]	−2.75 [−3.64, −1.87]	−1.33 [−1.86, −0.80]	−1.28 [−1.71, −0.85]	−1.30 [−1.81, −0.79]	−1.07 [−1.73, −0.42]	−2.80* [−3.52, −2.08]	−2.16 [−2.73, −1.59]
Model 3	−3.38 [−4.37, −2.40]	−2.39 [−3.37, −1.40]	−2.30 [−2.95, −1.66]	−2.39* [−3.61, −1.16]	−2.41* [−3.36, −1.46]	−2.75 [−3.64, −1.85]	−1.32 [−1.85, −0.79]	−1.28 [−1.71, −0.85]	−1.31 [−1.81, −0.80]	−1.06 [−1.72, −0.41]	−2.79* [−3.51, −2.08]	−2.16* [−2.73, −1.59]

Slopes (mm^3^/1.5 years) and 95 % Confidence Intervals were obtained from linear mixed models with the exposure fitted as tertiles. Model 1 adjusted for age, time, interaction term [time*independent variable (plant-sourced nitrate)]; Model 2 adjusted for all covariates in Model 1 plus physical activity levels, level of education, body mass index, smoking status, energy intake, marital status; Model 3 adjusted for all covariates in Model 2 excluding energy intake; plus, intake (yes/no) of alcohol, intake (g/d) of red meat, fish, processed meat, saturated fatty acids, polyunsaturated fatty acids, and monounsaturated fatty acids. Abbreviations: MRI, Magnetic Resonance Imaging; *APOE*, Apolipoprotein E (gene); n, number; mg/d, milligrams per day; median [Interquartile range]; g/day, grams per day.

⁎ , significant interaction between time and independent variable (*p* < 0.05).

⁎⁎ , significant interaction between time and independent variable (*p* < 0.01).

⁎⁎⁎ , significant interaction between time and independent variable (*p* < 0.001) in comparison of slopes of tertile 2 and 3 to tertile 1 .

In men *APOE* ε4 allele carriers, compared to those in the lowest intake tertile, participants in the highest tertile of plant-sourced nitrate had significantly lower rates of right hippocampal atrophy and grey matter atrophy (Model 2; Table 4 and Fig. 1**F &** 1**E**). Similar associations were observed for intake of vegetable-sourced nitrate (Model 2; **Supplementary Table 1** and **Supplementary Figure 1D & 1E**). There were no significant associations between intakes of plant- and vegetable-sourced nitrate and rates of left hippocampal and white matter atrophy and nor between intakes of animal-sourced nitrate and brain volume atrophy rates.

#### 3.2.4 Source of nitrate and brain structure volumes in men and women *APOE* ε4 allele non-carriers

In women *APOE* ε4 allele non-carriers, compared to those in the lowest intake tertile, participants in the highest tertile of plant-sourced nitrate and vegetable-sourced nitrate intake had a significantly lower rate of grey matter atrophy (Model 2; Table 4 and Fig. 1**H, Supplementary Table 1** and **Supplementary Figure 1G**). Participants in the highest tertile of animal-sourced nitrate intake had a significantly lower rate of right hippocampal atrophy compared to those in the moderate tertile (Model 2; **Supplementary Table 2** and **Supplementary Figure 2E**).

In men *APOE* ε4 allele non-carriers, participants in the highest tertile of plant-sourced nitrate intake had lower rates of left hippocampal and white matter atrophy compared to those in the moderate tertile (Model 2; Table 4 and Fig. 1**I &** 1**J**), participants in the highest tertile of vegetable-sourced nitrate intake had lower rates of left hippocampal atrophy (Model 2; **Supplementary Table 1** and **Supplementary Figure 1H**), and participants in the highest tertile of animal-sourced nitrate intake had a lower rate of left and right hippocampal atrophy compared those in the moderate tertile, (Model 2; **Supplementary Table 2** and **Supplementary Figure 2F & 2G**).

In all analyses, the addition of dietary confounders (model 3) to the multivariable adjusted model (model 2) did not attenuate the associations observed.

## 4 Discussion

In this prospective cohort study of older adults who were cognitively unimpaired at baseline, 554 participants with PET brain scans and 335 participants with MRI brain scans were followed for up to 126 months. We observed associations between plant-derived nitrate intake and cerebral Aβ deposition, particularly in women and *APOE* ε4 carriers, high-risk populations. While associations were observed for brain volume atrophy, these findings exhibited variability among the four subgroups, lacking discernible patterns in relation to sex and *APOE* ε4 allele carriage.

The results lend some support to the hypothesis that habitual plant nitrate intake may improve neuroimaging markers of AD [8]. Prior evidence supporting this potential benefit is, however, limited. A recent observational study in the Rotterdam cohort reported that although a higher intake of vegetable nitrate was associated with a lower risk of dementia, no associations with total brain volume, cerebral perfusion, and white matter hyperintensity volume were observed [41]. Results from studies of optimal dietary patterns such as the Mediterranean diet (MeDi), high in nitrate-rich green leafy vegetables, are also inconsistent. A recent systematic review of cross-sectional studies reported inconclusive or no association of MeDi with hippocampal volume [42]. Additionally, a cross-sectional study from the Australian Women's Healthy Ageing Project reported that adherence to MeDi was not associated with cerebral Aβ in women *APOE* ε4 allele carriers and non-carriers [43]. In contrast, three observational studies conducted in three cohorts, including the AIBL cohort used in the current study [44], have shown that MeDi adherence is associated with both less cerebral Aβ burden [45] and slower cerebral Aβ deposition over time [44,46]. Unlike the current study, slower cerebral Aβ associated with higher MeDi adherence was independent of *APOE* ε4 carrier status [44,47] . The RUSH Memory and Aging study of autopsied brain tissue (73 % women participants) reported that participants in the highest intake tertile of green leafy vegetables had less global AD pathology (summarised as neurofibrillary tangles of tau, and neuritic diffuse plaques containing Aβ) when compared to the lowest intake tertile [48]. The results from these cross-sectional studies and our longitudinal study reported herein highlight the need for further longitudinal studies investigating the association of dietary nitrate with AD-related neuroimaging biomarkers of brain health and AD risk.

Among the protective associations observed, the majority were for nitrate intake sourced from plants and vegetables, while associations with nitrate from animal sources appeared inconsistent. These findings contribute to the growing body of evidence suggesting that the positive and detrimental health effects of nitrate are influenced by its source. The positive effects of nitrate are through the conversion to nitrite and NO via the entero-salivary pathway [16]. The negative health effects of nitrate are linked to its potential to form NOCs [11]. It is hypothesised that the positive or negative health effects via these two distinct pathways is governed by source-dependent factors. The formation of NOCs is hypothesised to be inhibited in the presence of vitamin C, vitamin E, folate, and polyphenols in plants but increased in the presence of amines and heme iron in meats [49]. The acceptable daily intake (ADI) of nitrate is set at 0 to 3.7 mg/kg body weight (∼260 mg/ 70 kg adult) by the Science Committee for Food and the Joint Food and Agriculture Organization/ World Health Organization (WHO) Expert Committee on Food Additives (JECFA). Notably, the ADI guidelines do not differentiate between source of nitrate intake. The nitrate dose in clinical trials that have demonstrated improvement in cognitive function and cerebral blood flow ranged from 310 mg to 775 mg in acute studies [17,19] and 397 mg to 800 mg in chronic studies [19,[50], [51], [52]]. In the current study the median intake of vegetable-sourced nitrate was the highest in women *APOE* ε4 allele carriers (101 mg/d) compared to men *APOE* ε4 allele carriers (76 mg/d) and *APOE* ε4 allele non-carriers [men (90 mg/d) and women (85 mg/d)], it is still however, significantly lower than the ADI, and the nitrate dose (155 to 1104 mg/d) that showed improvements in cardiovascular biomarkers, cognition, and cerebral perfusion in clinical trials. As opposed to plant-sourced nitrate, intakes of animal-sourced nitrate excluding processed meat were lower in our cohort. Moreover, unlike sources of plant nitrate that have wide variation in nitrate content, the range of estimated nitrate content across the different animal sources is much smaller. Thus, caution should be exercised in ascribing the observed associations to nitrate as they may also be due to other co-existing components within the whole food.

Protective associations between plant-source nitrate intake and Aβ deposition were evident among the subgroups at elevated AD risk, women *APOE* ε4 carriers and non-carriers, as well as men *APOE* ε4 carriers. No such association was observed among men *APOE* ε4 non-carriers, who exhibit the lowest risk profile. The *APOE* gene codes the ApoE protein which primarily functions as a lipid transporter in the brain and periphery [7]. The ε4 allele substantially increases risk of AD [53] and the presence of the ε2 allele seems to be protective against AD compared to ε3 [54]. Notably, *APOE* genotype impacts AD pathology differently in women and men [55]. Although the results are mixed, studies have reported that women with at least one ε4 allele have higher rates of hippocampal atrophy with age in cognitively unimpaired participants, compared to men [56]. In addition, a study reported that there was widespread brain hypometabolism and cortical thinning in women *APOE* ε4 carriers compared to women *APOE* ε4 non-carriers, whereas men *APOE* ε4 carriers had small clusters of brain hypometabolism and regions of cortical thinning compared to men *APOE* ε4 non-carriers [57]. In addition, *APOE* ε4 carriers have early and faster rates of Aβ deposition than *APOE* ε4 non-carriers [58]. Consistent with these observations, our results suggest that there is a significant interplay between sex and genes when considering the impact of dietary nitrate intake on AD-related neuroimaging biomarkers of brain health. However, no discernible pattern was observed between intakes of plant- and vegetable-sourced nitrate and the rate of decline in brain structure volumes across both sexes and among *APOE* ε4 carriers and non-carriers. This may be attributed to limited statistical power to detect consistent associations.

There are some limitations to be considered while interpreting the results of the current study. We cannot deduce causality or neglect unmeasured confounding factors from our observational findings. Moreover*,* as this study utilised baseline dietary intake data, diet may have changed over the follow-up period of 126 months. Although our preliminary investigations in this cohort suggest that dietary habits remain mostly consistent over time in cognitively unimpaired individuals. Nevertheless, there is also the possibility of recall bias as the dietary data were self-reported; however, the impact of recall bias is minimised through the inclusion of only cognitively unimpaired participants at the time of dietary assessment, in the current study, which would circumvent recall bias due to memory impairment, and the dietary data should be regarded as estimates rather than accurate absolute intake measurements. Additionally, the estimation of nitrate intake relies on a database of the nitrate content of foods and, although this is a comprehensive database, the nitrate values applied are an estimate only. Furthermore, nitrate from water was not considered as we did not know the nitrate levels of the water consumed. Any such misclassification of dietary nitrate intake would most likely have attenuated the power to detect an association. Participants had a wide range of follow-up periods ranging from 0 month to 126 months, with a median of 18 months. In addition, given the number of statistical tests, we only focussed on clear and consistent associations in the current study. Future studies with robust follow-up periods are needed. Lastly, the study cohort is comprised of mostly highly educated Caucasians, which limits the generalisability of our findings, particularly as the outcomes have not been validated in an independent cohort.

There are, however, several aspects of our study which provide confidence in our findings. We have utilised a well-characterised cohort, thereby increasing the internal validity of our results. We have taken a conservative approach to analysis by controlling for a wide range of demographic variables and potential dietary confounders. The dietary data were collected using an instrument previously validated in epidemiological studies [26]. Furthermore, the latest comprehensive nitrate databases were used to estimate dietary nitrate intake from different food sources [9,28,30]. Finally, the mean nitrate intake in the current study is consistent with that observed in other cohort studies investigating associations of dietary nitrate with cardiovascular risk factors [[59], [60], [61]].

## 5 Conclusion

Our study observed associations of source-specific nitrate intake, especially plant-derived nitrate, with cerebral Aβ deposition and brain volume atrophy. In women and *APOE* ε4 allele carriers plant nitrate intake was associated with cerebral Aβ deposition, while findings for brain volume atrophy varied across subgroups, with no clear patterns regarding sex and *APOE* ε4 allele carriage. Further research in larger cohorts is needed to validate our results, determine sex-specific efficacious dietary nitrate doses, and understand the mechanisms underlying our results and whether dietary intake of plant-sourced nitrate could be incorporated into health policies to reduce risk of AD.

## CRediT authorship contribution statement

**Anjana Rajendra:** Writing – original draft, Software, Methodology, Investigation, Formal analysis, Conceptualization. **Nicola P. Bondonno:** Writing – review & editing, Supervision, Methodology, Investigation, Conceptualization. **Kevin Murray:** Writing – review & editing, Methodology. **Liezhou Zhong:** Writing – review & editing, Methodology. **Stephanie R. Rainey-Smith:** Writing – review & editing, Supervision, Methodology, Investigation, Conceptualization. **Samantha L. Gardener:** Writing – review & editing, Supervision, Methodology, Investigation, Conceptualization. **Lauren C. Blekkenhorst:** Writing – review & editing, Methodology. **Vincent Doré:** Writing – review & editing, Methodology. **Victor L. Villemagne:** Writing – review & editing, Methodology. **Simon M. Laws:** Writing – review & editing, Methodology. **Belinda M. Brown:** Writing – review & editing, Methodology. **Kevin Taddei:** Writing – review & editing, Methodology. **Colin L. Masters:** Writing – review & editing, Methodology. **Christopher C. Rowe:** Writing – review & editing, Methodology. **Ralph N Martins:** Writing – review & editing, Methodology. **Jonathan M. Hodgson:** Writing – review & editing, Supervision, Methodology, Investigation, Conceptualization. **Catherine P. Bondonno:** Writing – review & editing, Supervision, Methodology, Investigation, Conceptualization.

## Conflict of interest

The AIBL study (www.AIBL.csiro.au) is a consortium between Austin Health, CSIRO, Edith Cowan University, the Florey Institute (The University of Melbourne), and the National Ageing Research Institute. In-kind support has also been provided by Sir Charles Gairdner Hospital, Cogstate Ltd, Hollywood Private Hospital, The University of Melbourne, and St Vincent's Hospital. The study has received partial financial support from the Alzheimer's Association (US), the Alzheimer's Drug Discovery Foundation, an Anonymous foundation, the Science and Industry Endowment Fund, the Dementia Collaborative Research Centres, the Victorian Government's Operational Infrastructure Support program, the Australian Alzheimer's Research Foundation, the National Health, and Medical Research Council (NHMRC), and The Yulgilbar Foundation. Numerous commercial interactions have supported data collection and analyses.

## Appendix Supplementary materials

Image, application 1
