# Supplementary material for: Baseline habitual dietary nitrate intake and Alzheimer's Disease related neuroimaging biomarkers in the Australian Imaging, Biomarkers and Lifestyle study of ageing
Source: J Prev Alzheimers Dis. 2025 Apr 11;12(6):100161. doi: 10.1016/j.tjpad.2025.100161 (PMC12434242; doi:10.1016/j.tjpad.2025.100161)
Supplement: Supplementary file 1 [file mmc1.docx]

**Supplementary Table 1. Longitudinal associations between vegetable-sourced nitrate intake and MRI-based brain volumes**

|  | ***APOE* ε4 carriers** | | | | | | ***APOE* ε4 non-carriers** | | | | | | |
| --- | --- | --- | --- | --- | --- | --- | --- | --- | --- | --- | --- | --- | --- |
|  | **Women** | | | **Men** | | | **Women** | | | **Men** | | | |
| **Vegetable sourced nitrate** | **T1 n=22** | **T2 n=21** | **T3 n=21** | **T1 n=19** | **T2 n=19** | **T3 n=18** | **T1 n= 41** | **T2 n= 41** | **T3 n= 41** | **T1 n=31** | **T2 n=31** | **T3 n=30** |  |
| **Intake (mg/day)** | **34 (26, 43)** | **62 (59, 68)** | **101 (89, 118)** | **31 (24, 37)** | **48 (42, 59)** | **73 (66, 81)** | **28 (24, 35)** | **47 (42, 53)** | **79 (68, 104)** | **31 (27, 38)** | **54 (50, 60)** | **87 (80, 98)** |  |
| **Left Hippocampal volume** |  |  |  |  |  |  |  |  |  |  |  |  |  |
| Model 1 | -0.02  [-0.03, -0.00] | -0.02  [-0.04, -0.01] | -0.01  [-0.02, -0.00] | -0.02  [-0.04, -0.01] | -0.04  [-0.05, -0.03] | - 0.01  [-0.03, -0.00] | -0.02  [-0.02, -0.01] | -0.01  [-0.02, -0.01] | -0.01  [-0.01, -0.00] | -0.02  [-0.04, -0.01] | -0.01  [-0.03, -0.00] | -0.00^*^  [-0.01, 0.00] |  |
| Model 2 | -0.02  [-0.03, -0.00] | -0.02  [-0.04, -0.01] | -0.01  [-0.02, -0.00] | -0.02  [-0.04, -0.01] | -0.04  [-0.06, -0.03] | - 0.02  [-0.03, -0.00] | -0.02  [-0.02, -0.01] | -0.01  [-0.02, -0.01] | -0.01  [-0.01, -0.00] | -0.02  [-0.04, -0.01] | -0.01  [-0.03, -0.00] | -0.00^*^  [-0.01, 0.00] |  |
| Model 3 | -0.02  [-0.03, -0.00] | -0.02  [-0.04, -0.01] | -0.01  [-0.02, 0.00] | -0.02  [-0.04, -0.01] | -0.04  [-0.06, -0.03] | -0.02  [-0.03, -0.00] | -0.02  [-0.02, -0.01] | -0.01  [-0.02, -0.01] | -0.01  [-0.01, -0.00] | -0.02  [-0.04, -0.01] | -0.01  [-0.03, -0.00] | -0.00^*^  [-0.01, 0.00] |  |
| **Right hippocampal volume^*^** |  |  |  |  |  |  |  |  |  |  |  |  |  |
| Model 1 | -0.03  [-0.05, -0.02] | -0.02  [-0.03, -0.01] | -0.01^*^  [-0.02, -0.00] | -0.02  [-0.03, -0.00] | -0.04^*^  [-0.05, -0.03] | -0.01  [-0.02, 0.00] | -0.01  [-0.02, -0.01] | -0.01  [-0.02, -0.01] | -0.01  [-0.01, -0.00] | -0.01  [-0.02, -0.00] | -0.01  [-0.02, -0.00] | -0.01 [-0.01, -0.00] |  |
| Model 2 | -0.03  [-0.05, -0.02] | -0.02  [-0.03, -0.01] | -0.01^*^  [-0.02, -0.00] | -0.02  [-0.03, -0.00] | -0.04^**^  [-0.06, -0.03] | -0.01  [-0.02, -0.00] | -0.01  [-0.02, -0.01] | -0.01  [-0.02, -0.01] | -0.01  [-0.02, -0.00] | -0.01  [-0.02, -0.00] | -0.01  [-0.02, -0.00] | -0.01  [-0.01, -0.00] |  |
| Model 3 | -0.03  [-0.05, -0.02] | -0.02  [-0.03, -0.01] | -0.01^*^  [-0.02, -0.00] | -0.02  [-0.03, -0.00] | -0.04^**^  [-0.06, -0.03] | -0.01  [-0.02, 0.00] | -0.01  [-0.02, -0.01] | -0.01  [-0.02, -0.01] | -0.01  [-0.02, -0.00] | -0.01  [-0.02, -0.00] | -0.01  [-0.02, -0.00] | -0.01  [-0.01, -0.00] |  |
| **Grey matter volume^*^** |  |  |  |  |  |  |  |  |  |  |  |  |  |
| Model 1 | -3.39  [-4.82, -1.95] | -3.31  [-4.40, -2.22] | -2.23  [-3.22, -1.24] | -2.66  [-4.02, -1.29] | -4.83^*^  [-6.00, -3.66] | -2.21  [-3.51, -0.91] | 2.19  [-2.79, -1.59] | -2.64  [-3.07, -2.20] | -1.35^*^  [-1.91, -0.79] | -1.72  [-2.44, -1.00] | -1.42  [-2.18, -0.65] | -1.28  [-1.93, -0.62] |  |
| Model 2 | -3.31  [-4.76, -1.87] | -3.34  [-4.46, -2.23] | -2.31  [-3.33, -1.29] | -2.52  [-3.95, -1.09] | -4.91^*^  [-6.16, -3.66] | -2.20  [-3.53, -0.87] | -2.21  [-2.82, -1.61] | -2.71  [-3.16, -2.27] | -1.39^*^  [-1.94, -0.83] | -1.74  [-2.46, -1.01] | -1.49  [-2.29, -0.70] | -1.33  [-1.99, -0.66] |  |
| Model 3 | -3.26  [-4.69, -1.83] | -3.38  [-4.49, -2.28] | -2.40  [-3.43, -1.36] | -2.55  [-3.99, -1.11] | -4.91^*^  [-6.16, -3.67] | -2.16  [-3.49, -0.84] | -2.20  [-2.81, -1.60] | -2.71  [-3.16, -2.27] | -1.38^*^  [-1.94, -0.83] | -1.73  [-2.46, -1.01] | -1.49  [-2.28, -0.69] | -1.33  [-1.99, -0.66] |  |
| **White matter volume** |  |  |  |  |  |  |  |  |  |  |  |  |  |
| Model 1 | -3.39  [-4.41, -2.37] | -2.44  [-3.22, -1.67] | -2.14^*^  [-2.84, -1.43] | -2.21  [-3.23, -1.19] | -2.58  [-3.45, -1.71] | -2.77  [-3.75, -1.80] | -0.99  [-1.54, -0.43] | -1.38  [-1.78, -0.98] | -1.24  [-1.75, -0.72] | -1.64  [-2.28, -0.99] | -2.48  [-3.16, -1.79] | -2.03  [-2.62, -1.44] |  |
| Model 2 | -3.39  [-4.39, -2.38] | -2.41  [-3.19, -1.62] | -2.23  [-2.95, -1.52] | -2.14  [-3.21, -1.07] | -2.48  [-3.40, -1.56] | -2.89  [-3.87, -1.91] | -1.10  [-1.66, -0.53] | -1.44  [-1.85, -1.02] | 1.24  [-1.76, -0.73] | -1.60  [-2.25, -0.95] | -2.41  [-3.13, -1.70] | -1.99  [-2.59, -1.39] |  |
| Model 3 | -3.38  [-4.37, -2.39] | -2.45  [-3.21, -1.68] | -2.22  [-2.98, -1.46] | -2.20^*^  [-3.28, -1.12] | -2.51  [-3.43, -1.59] | -2.87  [-3.86, -1.89] | -1.10  [-1.67, -0.53] | -1.44  [-1.85, -1.03] | -1.25  [-1.76, -0.74] | -1.59  [-2.24, -0.94] | -2.41  [-3.13, -1.70] | -1.98  [-2.58, -1.39] |  |

Slopes (mm^3^/1.5 years) and 95% Confidence intervals were obtained from linear mixed models with the exposure fitted as tertiles. Model 1 adjusted for age, time, interaction term [time*independent variable (vegetable-sourced nitrate)]; model 2 adjusted for all covariates in model 1 plus physical activity levels, level of education, body mass index, smoking status, energy intake, marital status; model 3 adjusted for all covariates in model 2 excluding energy intake; plus, intake (yes/no) of alcohol, (g/d) of red meat, fish, processed meat, saturated fatty acids, polyunsaturated fatty acids, and monounsaturated fatty acids. Abbreviations: MRI, Magnetic resonance image; AD, Alzheimer’s disease; AIBL Australian Imaging, Biomarkers and Lifestyle study; *APOE*, Apolipoprotein E; n, number; T, tertile, mg/d; milligram per day, median (Inter quartile range); g/day, grams per day; ^*^, significant interaction between time and independent variable (p<0.05); ^**^, significant interaction between time and independent variable (p<0.01); ^***^, significant interaction between time and independent variable (p<0.001) in comparison of slopes of tertile 2 and 3 to tertile 1.

**Supplementary Table 2. Longitudinal associations between animal-sourced nitrate intake and MRI based brain volumes.**

|  | ***APOE* ε4 carriers** | | | | | | ***APOE* ε4 non-carriers** | | | | | |
| --- | --- | --- | --- | --- | --- | --- | --- | --- | --- | --- | --- | --- |
|  | **Women** | | | **Men** | | | **Women** | | | **Men** | | |
| **Animal sourced nitrate** | **T1 n=22** | **T2 n=21** | **T3 n=21** | **T1 n=19** | **T2 n=19** | **T3 n=18** | **T1 n= 41** | **T2 n=41** | **T3 n=41** | **T1 n=31** | **T2 n=31** | **T3 n=30** |
| **Intake (mg/day)** | **2 (1, 3)** | **8 (6, 9)** | **12 (10, 17)** | **2.6 (1.8, 3.3)** | **4.9 (4.5, 7.1)** | **9.5 (9, 10.7)** | **2 (1, 3)** | **6 (5, 7)** | **10 (9, 12)** | **2 (1, 3)** | **4 (4, 5)** | **10 (8, 11)** |
| **Left Hippocampal volume** |  |  |  |  |  |  |  |  |  |  |  |  |
| Model 1 | -0.05  [-0.06, -0.03] | -0.00^***^  [-0.02, 0.00] | -0.00^***^  [-0.02, 0.00] | -0.02  [-0.04, -0.00] | -0.02  [-0.04, -0.01] | - 0.04  [-0.05, -0.02] | -0.01  [-0.01, -0.00] | -0.01  [-0.02, -0.01] | -0.01  [-0.02, -0.01] | -0.00  [-0.01, 0.01] | -0.03^**^  [-0.05, -0.02] | -0.00  [-0.02, 0.00] |
| Model 2 | -0.05  [-0.06, -0.03] | -0.00^***^  [-0.02, -0.00] | -0.00^***^  [-0.02, 0.00] | -0.02  [-0.04, -0.00] | -0.02  [-0.04, -0.01] | - 0.04  [-0.06, -0.02] | -0.01  [-0.01, -0.00] | -0.01  [-0.02, -0.01] | -0.01  [-0.02, -0.01] | -0.00  [-0.01, 0.01] | -0.03^**^  [-0.05, -0.02] | -0.00  [-0.02, 0.00] |
| Model 3 | -0.05  [-0.06, -0.03] | -0.00^***^  [-0.02, 0.00] | -0.00^***^  [-0.02, 0.00] | -0.03  [-0.05, -0.01] | -0.02  [-0.04, -0.01] | -0.05  [-0.07, 0.03] | -0.01  [-0.01, -0.01] | -0.01  [-0.02, -0.01] | -0.01  [-0.02, -0.01] | -0.00  [-0.01, 0.01] | -0.03^**^  [-0.05, -0.02] | -0.00  [-0.02, 0.00] |
| **Right hippocampal volume** |  |  |  |  |  |  |  |  |  |  |  |  |
| Model 1 | -0.04  [-0.05, -0.02] | -0.02 ^*^  [-0.03, -0.01] | -0.01^**^  [-0.02, -0.00] | -0.02  [-0.04, -0.01] | -0.03  [-0.04, -0.02] | -0.01  [-0.03, -0.00] | -0.01  [-0.01, -0.00] | -0.02^*^  [-0.02, -0.01] | -0.01  [-0.02, -0.01] | -0.00  [-0.01, -0.00] | -0.02^**^  [-0.03, -0.01] | -0.00  [-0.01, -0.00] |
| Model 2 | -0.04  [-0.05, -0.02] | -0.02 ^*^  [-0.03, -0.00] | -0.01^**^  [-0.02, -0.00] | -0.02  [-0.04, -0.00] | -0.03  [-0.04, -0.02] | -0.01  [-0.03, -0.00] | -0.01  [-0.01, -0.00] | -0.02^*^  [-0.02, -0.01] | -0.01  [-0.02, -0.01] | -0.00  [-0.01, -0.00] | -0.02^**^  [-0.03, -0.01] | -0.00  [-0.01, -0.00] |
| Model 3 | -0.04  [-0.05, -0.02] | -0.02 ^*^  [-0.03, -0.00] | -0.01^**^  [-0.02, -0.00] | -0.02  [-0.04, -0.01] | -0.03  [-0.05, -0.02] | -0.02  [-0.03, -0.00] | -0.01  [-0.02, -0.01] | -0.02^*^  [-0.02, -0.01] | -0.02  [-0.02, -0.01] | -0.00  [-0.01, -0.00] | -0.02^**^  [-0.03, -0.01] | -0.00  [-0.01, -0.00] |
| **Grey matter volume** |  |  |  |  |  |  |  |  |  |  |  |  |
| Model 1 | -5.62  [-6.82, -4.42] | -1.59^***^  [-2.58, -0.59] | -2.28^***^  [-3.18, -1.38] | -3.76  [-5.22, -2.29] | -3.05  [-4.19, -1.92] | -3.52  [-4.91, -2.13] | -2.57  [-3.07, -2.07] | -1.98  [-2.48, -1.47] | -1.85  [-2.48, -1.47] | -1.17  [-1.95, -0.40] | -2.01  [-2.75, -1.26] | -1.27  [-1.90, -0.64] |
| Model 2 | -5.71  [-6.96, -4.46] | -1.58^***^  [-2.58, -0.58] | -2.28^***^  [-3.19, -1.37] | -3.64  [-5.29, -1.99] | -3.06  [-4.22, -1.91] | -3.50  [-5.00, -2.00] | -2.61  [-3.12, -2.11] | -2.03  [-2.54, -1.52] | -1.87  [-2.44, -1.29] | -1.20  [-1.98, -0.43] | -2.04  [-2.80, -1.28] | -1.34  [-1.99, -0.70] |
| Model 3 | -5.70  [-6.96, -4.43] | -1.64^***^  [-2.64, -0.65] | -2.30^***^  [-3.21, -1.39] | -4.01  [-5.68, -2.34] | -2.92  [-4.25, -1.60] | -4.25  [-6.04, -2.45] | -2.96  [-3.51, -2.40] | -2.55  [-3.14, -1.96] | -2.04  [-2.69, -1.39] | -1.19  [-1.97, -0.41] | -2.05  [-2.81, -1.29] | -1.34  [-1.99, -0.70] |
| **White matter volume** |  |  |  |  |  |  |  |  |  |  |  |  |
| Model 1 | -2.75  [-3.72, -1.78] | -2.12  [-2.93, -1.31] | -2.68  [-3.41, -1.96] | -2.53  [-3.60, -1.47] | -2.83  [-3.66, -2.01] | -2.10  [-3.10, -1.09] | -1.21  [-1.66, -0.76] | -0.87  [-1.33, -0.41] | -1.75  [-2.27, -1.23] | -1.36  [-2.05, -0.66] | -2.30  [-2.97, -1.63] | -2.27  [-2.84, -1.71] |
| Model 2 | -2.69  [-3.67, -1.70] | -2.13  [-2.91, -1.34] | -2.79  [-3.52, -2.07] | -2.47  [-3.65, -1.29] | -2.69  [-3.51, -1.87] | -2.26  [-3.34, -1.18] | -1.24  [-1.70, -0.79] | -0.94  [-1.41, -0.47] | -1.80  [-2.32, -1.28] | -1.32  [-2.02, -0.63] | -2.24  [-2.92, -1.57] | -2.21  [-2.79, -1.63] |
| Model 3 | -2.71  [-3.70, -1.72] | -2.15  [-2.93, -1.36] | -2.78  [-3.52, -2.03] | -2.63  [-3.82, -1.45] | -3.59  [-4.53, -2.65] | -2.29  [-3.59, -1.00] | -1.39  [-1.90, -0.88] | -1.09  [-1.64, -0.55] | -2.09  [-2.68, -1.49] | -1.32  [-2.01, -0.62] | -2.24  [-2.92, -1.57] | -2.21  [-2.79, -1.63] |

Slopes (mm^3^/1.5 years) and 95% Confidence intervals were obtained from linear mixed models with the exposure fitted as tertiles. Model 1 adjusted for age, time, interaction term [time*independent variable (animal-sourced nitrate)]; model 2 adjusted for all covariates in model 1 plus physical activity levels, level of education, body mass index, smoking status, energy intake, marital status; model 3 adjusted for all covariates in model 2 excluding energy intake; plus, intake (yes/no) of alcohol, (g/d) of saturated fatty acids, polyunsaturated fatty acids, monounsaturated fatty acids, and vegetables. Abbreviations: MRI, Magnetic resonance image; AD, Alzheimer’s disease, AIBL Australian Imaging, Biomarkers and Lifestyle study; *APOE*, Apolipoprotein E; n, number; T, tertile, mg/d; milligram per day, median (Inter quartile range); g/day, grams per day; ^*^, significant interaction between time and independent variable (p<0.05); ^**^, significant interaction between time and independent variable (p<0.01); ^***^, significant interaction between time and independent variable (p<0.001) in comparison of slopes of tertile 2 and 3 to tertile 1.


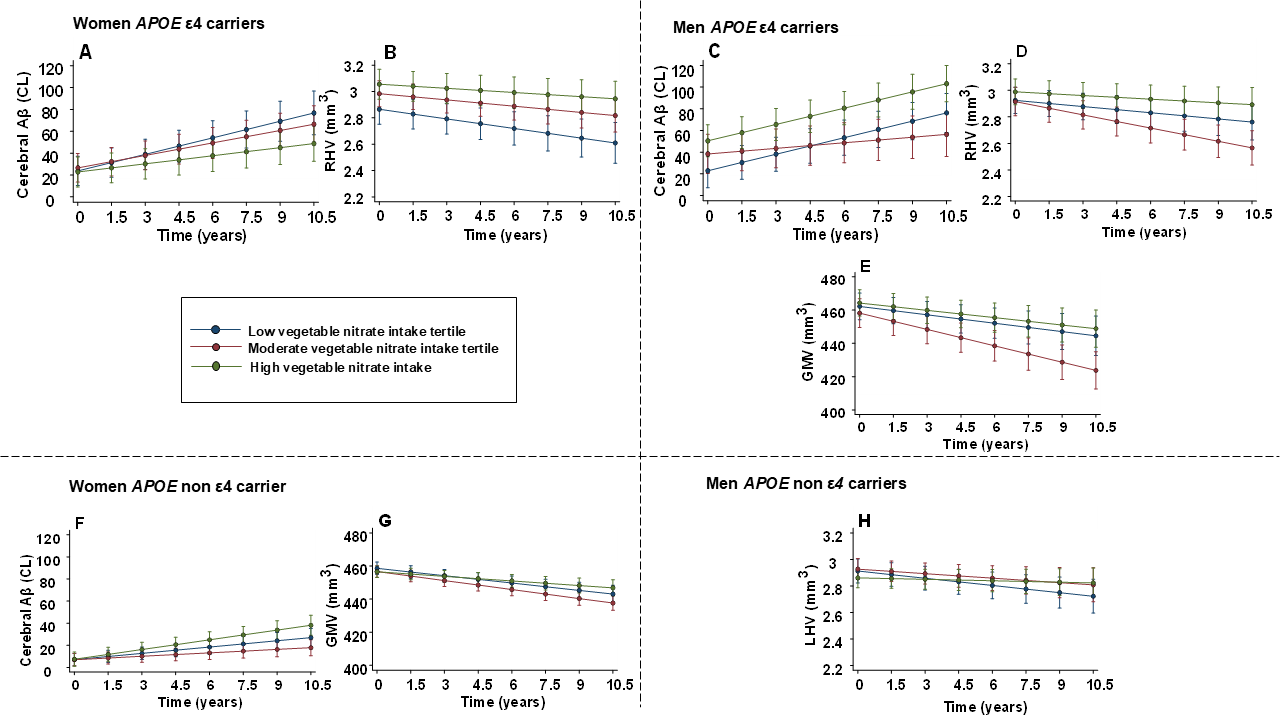


**Supplementary Figure 1. Trajectories of AD-related neuroimaging biomarkers of brain health by vegetable-sourced nitrate intake tertiles.** Interaction plots describing the associations between intakes of vegetable-sourced nitrate and rates of deposition of cerebral beta-amyloid and decline in volume of MRI-based biomarkers of AD in the Australian Imaging, Biomarkers and Lifestyle study of ageing followed up for 10.5 years. Plots based on linear mixed effects models adjusted for age, time, interaction term [time*independent variable (dietary nitrate)], physical activity levels, level of education, body mass index, smoking status, energy intake, marital status [Model 2]. Abbreviations: Abbreviations: AD, Alzheimer’s disease; *APOE*, Apolipoprotein E (gene); Aβ, beta-amyloid; CL, Centiloid; mm^3^ cubic millimetres; LHV, Left hippocampal volume; RHV, Right hippocampal volume; GMV, Grey matter volume.


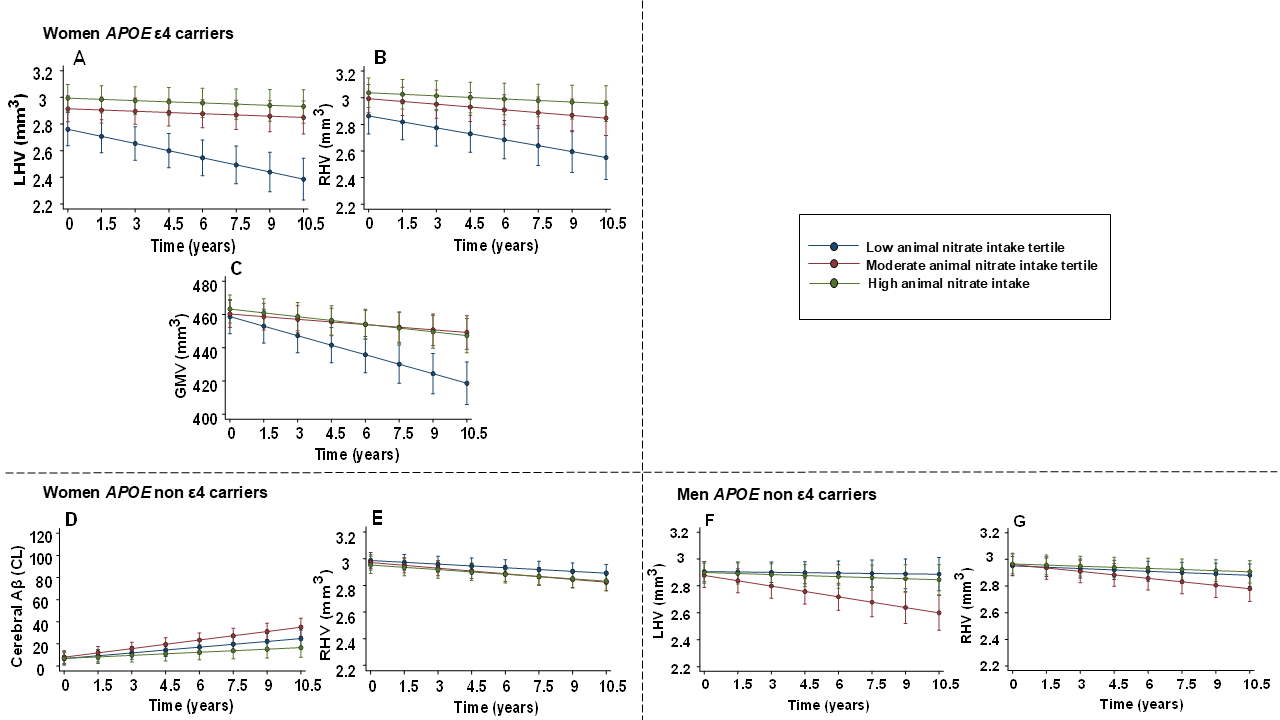


**Supplementary Figure 2. Trajectories of AD-related neuroimaging biomarkers of brain health by animal-sourced nitrate intake tertiles.** Interaction plots describing the associations between intakes of animal-sourced nitrate and rates of deposition of cerebral beta-amyloid and decline in volume of MRI-based biomarkers of AD in the Australian Imaging, Biomarkers and Lifestyle study of ageing followed up for 10.5 years. Plots based on linear mixed effects models adjusted for age, time, interaction term [time*independent variable (dietary nitrate)], physical activity levels, level of education, body mass index, smoking status, energy intake, marital status [Model 2]. Abbreviations: AD, Alzheimer’s disease; *APOE*, Apolipoprotein E (gene); Aβ, beta-amyloid; CL, Centiloid; mm^3^ cubic millimetres; LHV, Left hippocampal volume; RHV, Right hippocampal volume; GMV, Grey matter volume; WMV, White matter volume
